# Identification of Two *de novo* Variants of *CACNA1A* in Pediatric Chinese Patients With Paroxysmal Tonic Upgaze

**DOI:** 10.3389/fped.2021.722105

**Published:** 2021-09-24

**Authors:** Li-Ping Zhang, Yu Jia, Yu-Ping Wang

**Affiliations:** ^1^Department of Pediatric, Xuanwu Hospital, Capital Medical University, Beijing, China; ^2^Department of Neurology, Xuanwu Hospital, Capital Medical University, Beijing, China

**Keywords:** paroxysmal tonic upgaze, growth retardation, *CACNA1A* mutation, pediatrics, next-generation sequencing

## Abstract

**Objective:** Investigate the clinical manifestations and genotypes of paroxysmal tonic upgaze (PTU) in Chinese children.

**Patients and Methods:** We report the clinical manifestations and genetic test results of four pediatric PTU patients in China. Recent articles on PTU cases are also summarized and analyzed.

**Results:** The onset age of all four cases was at early infancy, and they presented as episodic binocular upward gaze with mild growth retardation. Two patients each carried a novel *de novo* variant in the *CACNA1A* gene, c.4046C>T (p.R1349X), and c.4415C>T (p.S1472L).

**Conclusion:** Patients with infantile-onset paroxysmal binocular upward gaze should be considered to diagnose as PTU.

## Introduction

Paroxysmal tonic upgaze (PTU, OMIM:168885) was pioneered by Ouvrier RA et al. in 1988. The article described an abnormal eye movement that occurred in early childhood. The disease presented as a transient, episodic, and involuntary upward movement of the eyes, with the chin typically held low. Forced nystagmus can also be observed during downward gaze. During episodes, horizontal eye movements stayed normal, and the patients remained conscious (1). PTU usually affected children under 2 years old, and it was characterized by diurnal fluctuations. The frequency of attacks decreased during sleep and increased during fever or fatigue (2). The benign course in which the symptoms usually resolve spontaneously within 12 months after onset has been reported in two-thirds of pediatric patients, while a small proportion of patients have a poor outcome in which symptoms persist, especially for developmental delay or ataxia (3).

To date, mutations in *CACNA1A, GRID2*, and *SEPSECS* genes have been identified to be associated with PTU (4–6). Mutations in *ADAMTS2, CACNA1A, CDK13, SIM1*, and *ZNF331* have also been reported as PTU-associated in the Human Gene Mutation Database (HGMD) (7), while only two mutations in *CACNA1A* are clearly classified as disease-causing (5). There is still a gap between PTU and its genetic background. Herein, we performed next-generation sequencing on four pediatric PTU patients and identified two novel *de novo CANA1A* variants. In this study, we report the clinical manifestations and genetic information of these patients to discuss PTU and its causative variants.

## Methods

Informed consent for genetic testing was obtained from all patients and their families and reviewed by the Xuanwu hospital's ethics committee. Peripheral blood samples of patients and their parents were collected and sent to Running Gene Inc. (Beijing, China) for whole-exome sequencing. Genomic DNA was extracted from blood samples following the instruction of Blood DNA Kit V2 (Cwbio, China, CW2553). Qualified DNA samples were fragmented into 200–300 bp. DNA libraries were prepared with the KAPA Library Preparation Kit (Kapa Biosystems, KR0453). Then, pooled libraries were hybridized by IDT and xGen Lockdown® Probes (Integrated DNA Technologies, USA) to capture target fragments. Captured libraries were then sequenced on the Illumina Novaseq (Illumina, CA, USA) as paired-end 150-bp reads. Raw data (stored in FASTQ format) were collected, qualified, and filtered. Qualified reads were aligned to the human reference genome sequence GRCh37 hg19 using Burrows–Wheeler Alignment tool (8). Consensus single-nucleotide polymorphisms (SNP) and insertions and deletions (indels) are called using GATK (9). All the called variants were annotated using public databases (1,000 genomes project, ExAC, gnomAD, Ensembl, etc.). The annotation content helps to locate disease-associated variants. Candidate variants were analyzed based on the American College of Medical Genetics (ACMG) guidelines (10). Sanger sequencing was also performed to verify the segregation of candidate variants in their families. DNA paternity testing was performed on the families of patients 1 and 4.

## Results

### Case Presentation

Patient 1 is a 6-year-old girl who was admitted to our hospital due to episodic binocular upward gaze for more than 4 years. No specific perinatal history and family history were reported for the patient. She could talk and walk independently at 2 years old but could not walk steadily and easily fell. The girl is educated in primary school but she has a poor performance. The paroxysmal slanting neck appeared at 3 months after birth without any facial color change nor body movement. Each attack lasted for several minutes, and the patient was conscious during the attack. However, the symptom disappeared after the age of 2 years. Episodic binocular upward gaze occurred just before the age of 2 years, with no obvious cause. It manifested as a sudden upward gaze with a head-down and a dull gaze while awake for 5–10 s per episode. Each episode occurred with or without body weakness but without falling over. More than 10 episodes were observed per day, with a higher frequency during fever, exertion, or a supine position. Her general state during the interictal period was in good condition. She also had febrile seizures, with about 10 times seizures at the age of 1.5 years. She was diagnosed with epilepsy in another institution and was given levetiracetam, sodium valproate, nimetazepam, and topiramate. All of the treatments were ineffective. She took only levetiracetam oral solution 2 ml/day at the examination.

**Figure 1:**
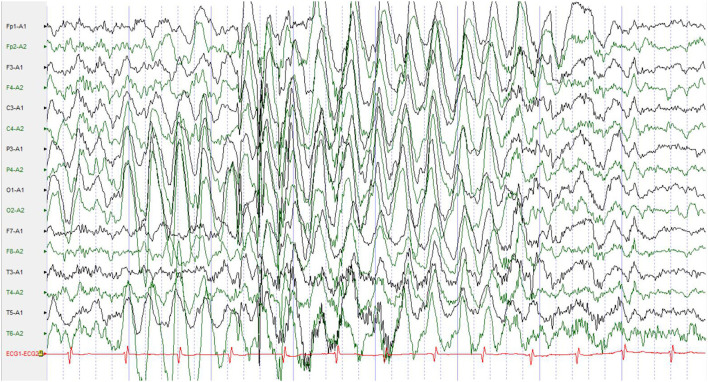
No abnormalities were found in the EEG during episodes, but all-conductor paroxysmal high to very high amplitude slow waves were seen in the interictal awake and sleep period, with rhythmic distribution, mainly in the bilateral posterior head. EEG, electroencephalogram.

Additional examinations: Biochemical test, routine blood test, and brain magnetic resonance imaging (MRI) showed no abnormalities. No abnormalities were found in the electroencephalogram (EEG) during episodes, but all-conductor paroxysmal high to very high amplitude slow waves were seen in the interictal awake and sleep period, with rhythmic distribution, mainly in the bilateral posterior head (Figure 1).

Patient 2 is a 1.5-year-old boy. He was admitted to our hospital due to an episodic binocular upward gaze for nearly 1 year. No specific perinatal history and family history were reported for the patient. He could hold his head up at 3 months, sit alone at 8 months, and walk alone at 1 year and 3 months. He could say single words at the examination. The paroxysmal slanting neck appeared at 6 months after birth and resolved spontaneously several months later. Binocular upward gaze with head down appeared at 7 months after birth. It usually struck the boy every 2–3 days, for 1–2 s per episode. He had a maximum of three episodes in 1 day and no episodes for up to 10 days. The attacks had nothing to do with fever and infection. His state was generally good in the interictal period. His parents refused to take any drugs.

Additional examination: Brain MRI and EEG showed normal.

Patient 3 is a 6-year-old boy. He was admitted to our hospital due to episodic binocular upward gaze for more than 5 years. The patient's hands and feet were bruised 3 days after birth. He was diagnosed with a subarachnoid hemorrhage at that time. He had lack of stability and was unable to walk in a straight line at physical examination. He also had delay in growth and development. No specific family history was reported. The paroxysmal slanting neck appeared at 1 month after birth and resolved spontaneously several months later. Episodic binocular upward gaze appeared at 3–4 months after birth. Sometimes there were episodes of head down, mouth opening, or head tilting back or to the side. During the attack, the patient was conscious and responsive, and he could be relieved in 3–5 s. However, episodes occurred continuously, and a series of episodes lasted 10–20 min. Episodes occurred daily, or a series of episodes occurred every 2 weeks. The attacks were not associated with fever and infection. Madopar at 3.125 mg once a day was given to the patient, and his symptom improved slightly after 2 weeks.

**Figure 2:**
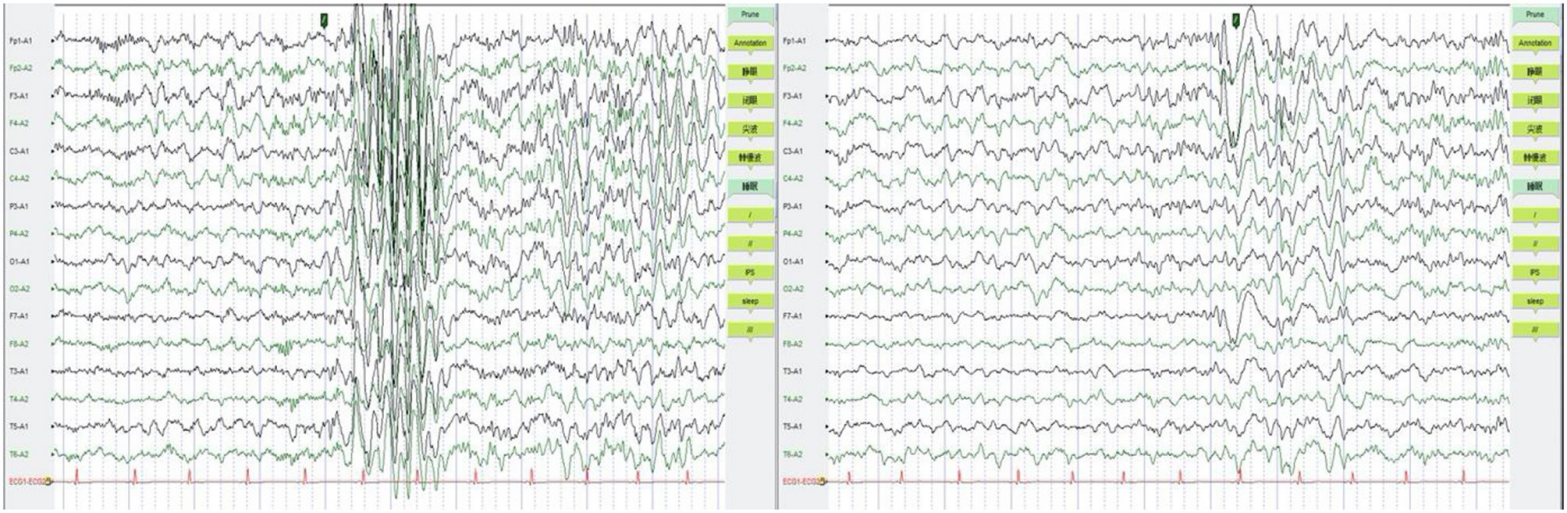
EEG were shown to be normal during episodes, but occasional short-range paroxysms of medium to high amplitude spike waves were seen during awake period (left). Medium to high amplitude spike-wave emissions were observed in the left anterior head during sleep (right). EEG, electroencephalogram.

Additional examinations: No abnormalities were found in brain MRI. EEG showed normal result during episodes, but occasional short-range paroxysms of medium to high amplitude spike waves were seen during awake periods. Medium to high amplitude spike-wave emissions were observed in the left anterior head during sleep (Figure 2).

Patient 4 is an 8-month-old girl who has episodic binocular upward gaze for 7 months. She is delayed in growth development. She raised her head at 4 months but could not sit alone steadily until the present. The Children's Developmental Center of China scores were 78/81 for her (normal reference >90 in both movement and language). No specific perinatal history and family history was reported. The paroxysmal slanting neck was present after birth. Episodic upward gaze appeared 1 month after birth, presenting with an upward turning of the eyes and a slight bowing of the head, without physical movements, and relieved in about 10 s. The episode occurred daily after waking up, and the recurrent episodes lasted up to several hours. The patient's general state was good during the interictal period. No evidence showed the episodes were related to fatigue or infection. The patient took valproic acid for a few days but discontinued it due to ineffectiveness. She was also given methocarbamol 0.025 g twice a day orally, which was discontinued due to exacerbation of episodes. She took topiramate 2.5 mg 2 times a day before the examination, but with inefficacy.

**Figure 3:**
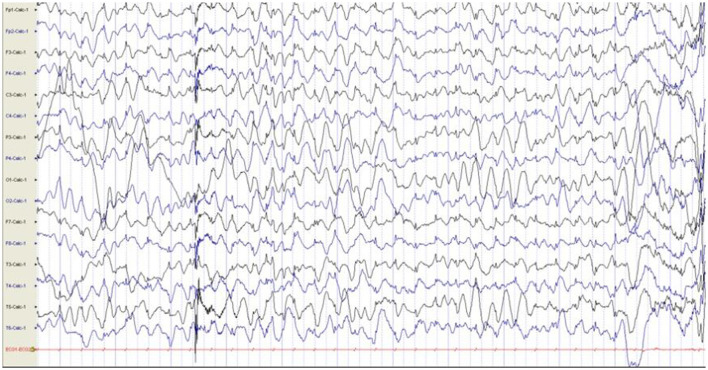
EEG showed interictal slow waves in the posterior brain, and no abnormalities were found in the EEG during episodes. EEG, electroencephalogram.

Additional examinations: The patient can chase sounds and objects and roll over but cannot sit alone steadily nor crawl at physical examination. Brain MRI was normal, EEG showed interictal slow waves in the posterior brain, and no abnormalities were found in the EEG during episodes (Figure 3).

A summary of the clinical characteristic of our four patients with PTU is shown in Table 1.

**Table 1:** Clinical characteristic of our patients with paroxysmal tonic upgaze.

**Number**	**Gender**	**PTU onset age**	**Paroxysmal slanting neck**	**PTU last time**	**PTU frequency**	**Promotor factors**	**EEG**	**MRI**	**Development**	**Gene**	**Effective drug**
Patient 1	Female	Before 2 years old	Yes	5–10 s	10 episodes per day.	Fever, exertion, or a supine position	Abnormal	Negative	Delay	*CACNA1A*	Madopar
Patiernt2	Male	7 months	Yes	1–2 s	A maximum of 3 episodes in 1 day and no episodes for up to 10 days.	No	Normal	Negative	Delay	Negative	No
Patient 3	Male	3–4 months	Yes	3–5 seconds	Episodes occurred daily or a series of episodes occurred every 2 weeks.	No	Abnormal	Negative	Delay	Negative	Madopar
Patient 4	Female	1 month	yes	10 s	Recurrent episodes lasted up to several hours every day.	No	Abnormal;	Negative	Delay	*CACNA1A*	No No

### Next-Generation Sequencing

**Figure 4:**
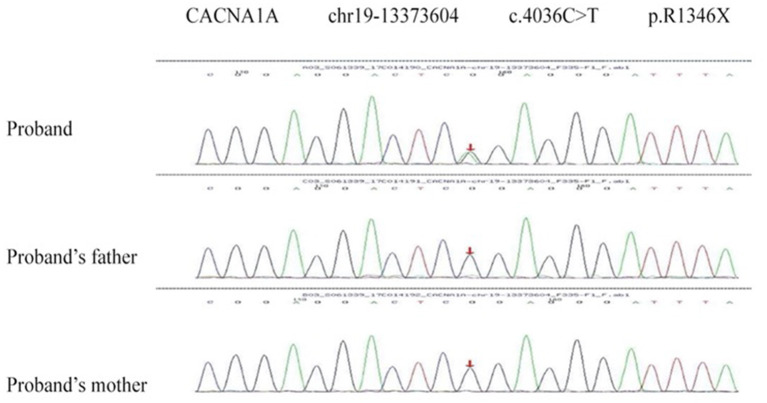
*De novo* variant in *CACNA1A* (NM_023035), c.4046C>T (p.R1349X) in patient 1.

**Figure 5:**
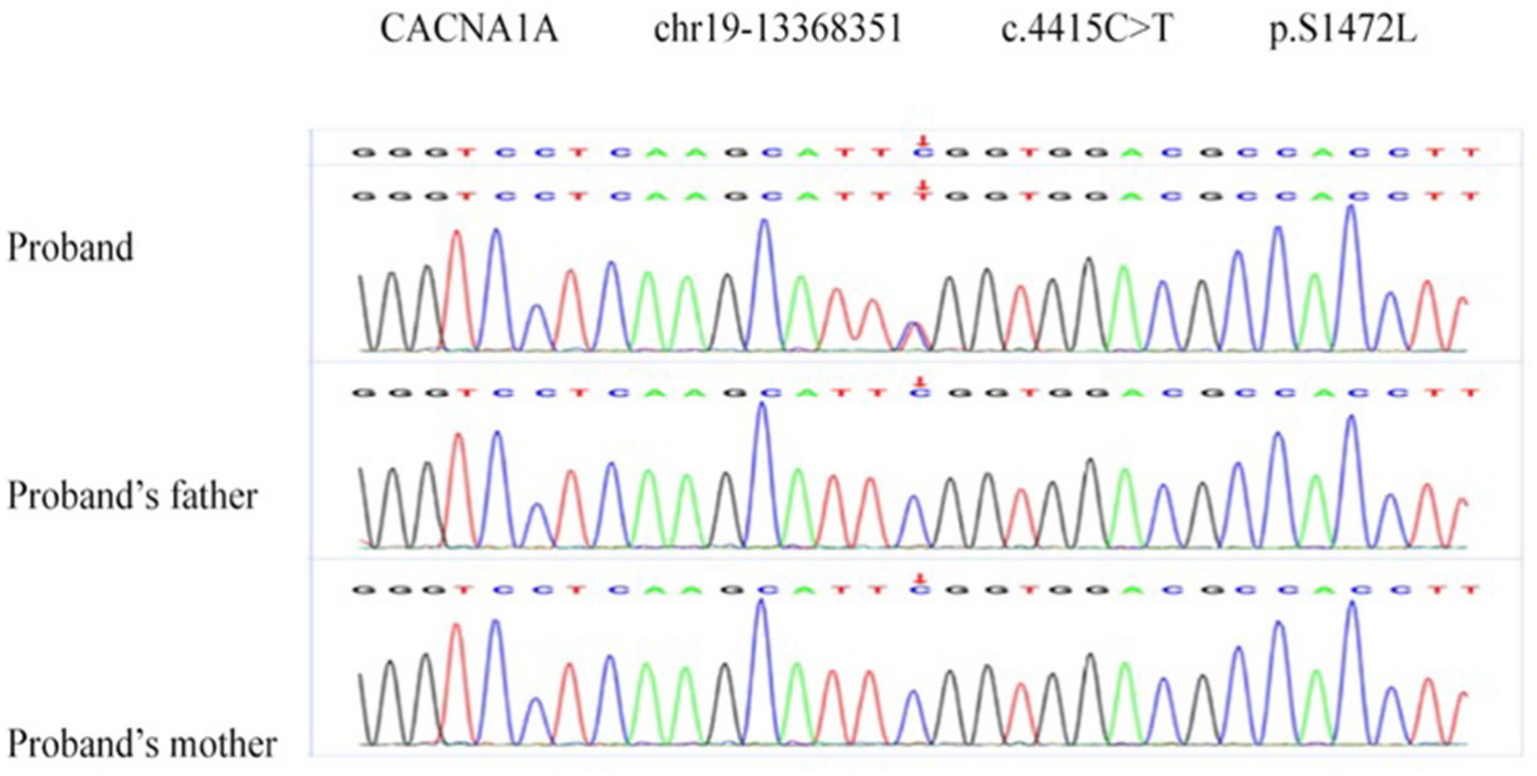
*De novo* variant in *CACNA1A* (NM_023035), c.4415C>T (p.S1472L) in patient 2.

Two *de novo* variants were identified in *CACNA1A* (NM_023035): c.4046C>T (p.R1349X) in patient 1 (Figure 4) and c.4415C>T (p.S1472L) in patient 4 (Figure 5). Both variants have not been reported in any database, so they are considered novel variants. No PTU-associated mutations were found in patients 2 and 3.

R1349X is a non-sense variant, probably causing a truncated protein or protein loss by non-sense-mediated decay (PVS1). The variant was never found in public databases (ExAC, 1000G, genmAD, and dbSNP) (PM2). The variant is assumed *de novo* because both parents carried wild type alleles at the variant site but without paternity and maternity confirmation (PM6). *In silico* computational predictions of R1349X showed disease causing (possibility: 1.000) in MutationTaster2 (11) and damaging (confidence score: 0.858) in SIFT (12) (PP3). According to the ACMG guidelines, c.4046C>T (p.R1349X) is considered pathogenic.

S1472L is a missense variant detected in *CACNA1A*. It was absent in the control database (PM2) and assumed *de novo*, without paternity and maternity confirmation (PM6). The variant is predicted to be disease causing (possibility: 1.000) in MutationTaster2, damaging (0, cutoff = 0.05) in SIFT, deleterious (−5.96, cutoff = −2.5) in Provean (13), and probably damaging with a score of 1.000 in Polyphen-2 (14) (PP3). Polyphen-2 and PhastCons (15) showed the mutant site is highly conserved across species. Thus, the variant c.4415C>T (p.S1472L) is assessed as VUS (variant of uncertain clinical significance).

## Discussion

The onset age of PTU is wide, ranging from the first week of life to 9 years old, but it usually affects infants and children under 2 years old. The onset is usually insidious, following a febrile illness or vaccination sometimes. The duration and frequency of episodes are variable. With time passing by, symptoms usually relieve and completely cease (16). In our cases, all patient's onset PTU are in infancy. The earliest one occurred 1 month after birth, and the latest onset was at nearly 2 years old. All of them had benign paroxysmal torticollis and resolved spontaneously after a few months.

The clinical features of PTU episodes were reviewed and summarized. The duration of PTU episodes is generally in the range from 3 s to 2 h, but there are also longer cases. The frequency of PTU episodes ranges from 2 to 3 per day to more than 10 per day. In some patients, fever, fatigue, stressful events, and supine position can exacerbate the frequency of episodes occurrence. Some cases may have a paroxysmal slanting neck before the onset of disease. More than one-third of patients exhibit coordination disorders (manifesting as ataxia) during recurrent PTU episodes. In a small number of children, coordination disorders persisted or reappeared during fever after the disappearance of PTU episodes. Brain MRI was normal in general. The episodes disappear spontaneously in most cases, although transient relapses have been reported. Some patients have developed sequelae of cognitive impairment, speech problems, and oculomotor problems such as nystagmus (17). All patients eventually improved and recovered from their symptoms (3). Our study is consistent with previous clinical and laboratory diagnosis of PTU. All four subjects have observed developmental delay, including head lag, late sitting up alone, delayed speech, poor numeracy, poor fine motor skills, and ataxia. Their onset was typical paroxysmal tonic upward gaze in infancy. Besides, episodes occur more frequently in the supine position, and the frequency of episodes increased with fever in one of our patients.

The etiology of PTU is still unclear, and there are four main pathogeneses: the immature brainstem, neurotransmitter depletion, immune mechanisms, and genetic disorders. In the seven PTU patients reported by Quade et al., three of the six children had normal MRI examinations and the remaining three showed non-specific abnormalities. Since none showed pathological brainstem changes, brainstem injury was not considered to be the cause of the disease (4). A few studies have reported abnormal brain MRI results in PTU patients, showing hypomyelination, delayed myelination, or lesions within bilateral mesencephalon and thalamus on brain MRI (17, 18). Nevertheless, the majority of affected children have normal MRI performance. Quade et al. also applied the cerebrospinal fluid test in two affected children. No abnormal findings have been found in biogenic amines, pterin, and folate examinations, which did not support the cause of neurotransmitter depletion (4). A previous study suggested that vitamin B_12_ deficiency might cause PTU and could have a corroboration of metabolic abnormalities as an etiology (19). Besides, a history of fever or vaccination before the onset of the disease in some patients may suggest an association with the immune event. In one case, transient PTU occurred during an upper respiratory tract infection, supporting the immunological mechanism. However, immunosuppression was also found to be ineffective in another patient (4). All of our four cases had normal MRI findings, which did not support a structural etiology. None of them had their cerebrospinal fluid examined, and only patient 1 had an increased frequency of episodes during infection, which was not seen in the other three cases and which indicated that immune mechanisms may not be an important factor.

Mutations in *CACNA1A* gene has been identified to associate with PTU previously. Mutations in this gene are associated with familial hemiplegic migraine, spinal cerebellar ataxia type 6, and ataxia type 2 (EA2) (5, 20). Roubertie et al. reported *CACNA1A* gene mutation in two generations of a big family. Several cases in the family had periodic paroxysmal neurological manifestations, which are consistent with the clinical description of PTU, benign paroxysmal torticollis of infancy (BPTI), or episodic ataxia (21). Lerman-Sagie et al. reported three cases of PTU caused by mutations in the *CACNA1A* gene, accompanied by motor and speech delay and cerebellar ataxia (5). Tantsis et al. reported three PTU-affected children with mutations in the *CACNA1A* gene who later developed hemiplegic migraine (20). The *CACNA1A* gene is located on chromosome 19 and encodes the alpha-1 subunit of the voltage-gated calcium channel, which is specifically expressed in the cerebellum. Our findings confirm *CACNA1*A mutations as an important cause of PTU and might expand the spectrum of *CACNA1A*-related diseases. A comparison of the clinical features of our patients with *CACNA1A*-related PTU with other series is shown in Table 2.

**Table 2:** Comparison of the clinical features of our patients with CACNA1A related PTU with other series.

**Variables**	**2 patients from our cases series**	**3 patients from Blumkin et al. (5) and 2 patients from Roubertie et al. (21)**
Age at onset	1 months—before 2 years	3 days-10 months
Duration of attacks	5–10 s	Clusters of 2–8 s lasting a few minutes to one hour
Frequency	More than 10 episodes per day/recurrent episodes lasted up to several hours.	Multiple daily episodes to several times per week
Triggers	Fever, exertion, supine position/or no triggers	Febrile illnesses, fatigue, supine position
Imagining of brain	Brain MRI normal	Brain MRI normal
PTU evolution	Recovery by 2 months after treatment/follow-up	Recovery by 13 months-5 years
Association ictal symptoms	Head-down, dull gaze, with or without body weakness/slight bowing of head	Ataxia, torticollis, pallor, chin down, downbeat nystagmus, side-to-side head movements, stereotypic hand movements
Association interictal disorders	Ataxia, paroxysmal torticollis, developmental delay	Ataxia, dystonia, dyskinesia, nystagmus, episodic coma, paroxysmal torticollis, developmental delay
Symptoms after PTU recovery	Cerebellar dysfunction: gait and limb ataxia/follow-up	Cerebellar dysfunction: gait and limb ataxia, nystagmus, abnormal smooth pursuit, dysarthria

PTU is currently considered to fall within the spectrum of dystonia disorders, an underlying cerebellar dysfunction resulting from a channelopathy. No significant efficacy of antiepileptic drugs and pro-adrenocorticosteroids has been reported. Levodopa is effective in some patients (22). A recent study reported that PTU patients have shown substantial clinical amelioration by acetazolamide treatment. Quade et al. reported a significant curative effect of using carbonic anhydrase inhibitors (three acetazolamide; two sulforaphane) in five patients with PTU, one of whom was a 3.5-year-old girl who suffered from PTU. Her mother was also suffering from episodic ataxia as well as myasthenia gravis, primary biliary cirrhosis, and severe attention deficiency, and her maternal aunt showed severe attention deficiency, too. Acetazolamide therapy resulted in marked clinical improvement in her and her mother's ataxic symptoms. Whereas, exome sequencing and sanger sequencing of the *CACNA1A* gene as well as Array-comparative genomic hybridization (CGH) analysis failed to identify disease-causing mutations, multiplex ligation-dependent probe amplification (MLPA) analysis finally proved a heterozygous deletion of exon 31 of the *CACNA1A* gene in the patient, her mother, her grandmother, and her maternal aunt (4).

In our cases, patient 1 took Madopar tablets 3.125 mg once a day after diagnosis; the frequency of episodes was reduced by 30% after half a month of the administration. Then the dosage was increased to 3.125 mg 2 times a day, and the frequency of episodes was reduced by more than half after over 1 month of treatments. Significant clinical effects were achieved at 2 months of administration with basically no episodes. Similarly, the symptom of patient 3 improved to some extent after the addition of 3.125 mg Madopar once a day. However, Madopar has worsened the conditions of patient 4, and thus the drug was discontinued. Currently, patient 4 is treated with acetazolamide, and the efficacy has to be observed. Further studies are needed to widen the view of PTU in Chinese children.

## Conclusion

PTU usually affected children under 2 years of age. Patients usually had benign paroxysmal torticollis before PTU attacks. *CACNA1A* gene might be a causative gene in PTU patients.

## Data Availability Statement

The data presented in the study are deposited in the Genome Sequence Archive for Human (https://ngdc.cncb.ac.cn/gsa-human/), with accession number PRJCA006618.

## Ethics Statement

The studies involving human participants were reviewed and approved by Xuanwu hospital. Written informed consent to participate in this study was provided by the participants' legal guardian/next of kin. Written informed consent was obtained from the individual(s), and minor(s)' legal guardian/next of kin, for the publication of any potentially identifiable images or data included in this article.

## Author Contributions

L-PZ was the major contributor in writing the manuscript. L-PZ and Y-PW contributed to the diagnosis and treatment of the patients. YJ contributed to the analysis of genes. Y-PW contributed to checking the manuscript. All authors read and approved the final manuscript.

## Funding

This work was supported by the National Natural Science Foundation of China (Grant No. 81771398), Beijing Key Clinical Speciality Excellence Project, National Support Provincial Major Disease Medical Services, and Social Capability Enhancement Project.

## Conflict of Interest

The authors declare that the research was conducted in the absence of any commercial or financial relationships that could be construed as a potential conflict of interest.

## Publisher's Note

All claims expressed in this article are solely those of the authors and do not necessarily represent those of their affiliated organizations, or those of the publisher, the editors and the reviewers. Any product that may be evaluated in this article, or claim that may be made by its manufacturer, is not guaranteed or endorsed by the publisher.
